# Tolerance of *Yarrowia lipolytica* to inhibitors commonly found in lignocellulosic hydrolysates

**DOI:** 10.1186/s12866-021-02126-0

**Published:** 2021-03-08

**Authors:** Oliver Konzock, Simone Zaghen, Joakim Norbeck

**Affiliations:** Department of Biology and Biological Engineering, division of Systems and Synthetic Biology, Chalmers University of Technology, Göteborg, Sweden

**Keywords:** *Yarrowia lipolytica*, Lignocellulosic biomass, Inhibitor tolerance, Xylose utilization, Furfural, HMF, Cinnamic acid, Coniferyl aldehyde, Formic acid, Acetic acid

## Abstract

**Background:**

Lignocellulosic material is a suitable renewable carbon and energy source for microbial cell factories, such as *Yarrowia lipolytica*. To be accessible for microorganisms, the constituent sugars need to be released in a hydrolysis step, which as a side effect leads to the formation of various inhibitory compounds. However, the effects of these inhibitory compounds on the growth of *Y. lipolytica* have not been thoroughly investigated.

**Results:**

Here we show the individual and combined effect of six inhibitors from three major inhibitor groups on the growth of *Y. lipolytica*. We engineered a xylose consuming strain by overexpressing the three native genes XR, XDH, and XK and found that the inhibitor tolerance of *Y. lipolytica* is similar in glucose and in xylose. Aromatic compounds could be tolerated at high concentrations, while furfural linearly increased the lag phase of the cultivation, and hydroxymethylfurfural only inhibited growth partially. The furfural induced increase in lag phase can be overcome by an increased volume of inoculum. Formic acid only affected growth at concentrations above 25 mM. In a synthetic hydrolysate, formic acid, furfural, and coniferyl aldehyde were identified as the major growth inhibitors.

**Conclusion:**

We showed the individual and combined effect of inhibitors found in hydrolysate on the growth of *Y. lipolytica*. Our study improves understanding of the growth limiting inhibitors found in hydrolysate and enables a more targeted engineering approach to increase the inhibitor tolerance of *Y. lipolytica*. This will help to improve the usage of *Y. lipolytica* as a sustainable microbial cell factory.

**Supplementary Information:**

The online version contains supplementary material available at 10.1186/s12866-021-02126-0.

## Introduction

*Yarrowia lipolytica* is an oleaginous yeast that can naturally produce more than 20% of its dry cell weight as storage lipids. Through genetic engineering and growth conditions optimization, the lipid content can be increased up to 80% [1]. *Y. lipolytica* is increasingly used as a host for lipid-derived products [1], but also other products, e.g. plant natural products [2]. However, most bioprocesses are based on first-generation biomasses (e.g. starch and sugars from corn or wheat) which compete with food production and which are expensive, contributing up to 60% of the total cost of a bioprocess [3]. Therefore, both from an environmental and economical perspective, switching to less expensive carbon sources that do not compete with food production would be highly desirable. Lignocellulosic biomass is such an alternative carbon source, which is usually derived from agricultural waste or forestry residues.

Lignocellulose is a comparatively cheap and abundant resource. It mainly consists of lignin, which has a structural and protective function, cellulose and hemicellulose. Cellulose is a polysaccharide of glucose, while hemicellulose is mainly made of arabinose, galactose, glucose, mannose, and xylose [4]. The most abundant sugar in hemicellulose is usually xylose [5], although mannose is the most abundant sugar in softwood [6]. *Y. lipolytica* can naturally utilize many of these sugars, but xylose utilization in *Y. lipolytica* usually requires genetic engineering [7].

Since microorganisms, such as *Y. lipolytica* cannot utilize the untreated lignocellulosic biomass, a hydrolysis pretreatment is required to release the sugar monomers from the polymers. Most hydrolysis methods involve applying high pressure and/or high temperatures on the biomass in combination with strong acids or bases, often also combined with enzymatic treatments, as reviewed by [4]. During the pre-treatment, several compounds with inhibitory effects are formed, which can mainly be divided into three main groups: furanic aldehydes, weak acids, and aromatic compounds [8], but depending on the process, other classes of inhibitory compounds can also occur. There are two major ways to deal with this problem, the first being chemical modification or removal of the inhibitors, the second being the use or development of microorganisms with inherent tolerance to those inhibitors [4].

The most studied hydrolysate inhibitors are the furanic aldehydes, furfural (2-furaldehyde) and HMF (5-Hydroxymethylfurfural). HMF is formed by the dehydration of hexoses, while furfural is formed by the dehydration of pentoses. The inhibitory mechanisms of furfural and HMF are to a large extent due to their reactive aldehyde groups, which produce reactive oxygen species (ROS) that cause DNA mutations, protein misfolding, and membrane damage [9]. The repair of these damages causes a reduction in the intracellular levels of ATP, NADH, and NADPH which in turn results in growth inhibition and a prolonged lag phase [10]. Furthermore, furfural and HMF are thought to inhibit key enzymes of cellular metabolism, e.g. two glycolytic enzymes hexokinase and glyceraldehyde-3-phosphate dehydrogenase [11].

**Fig. 1:**
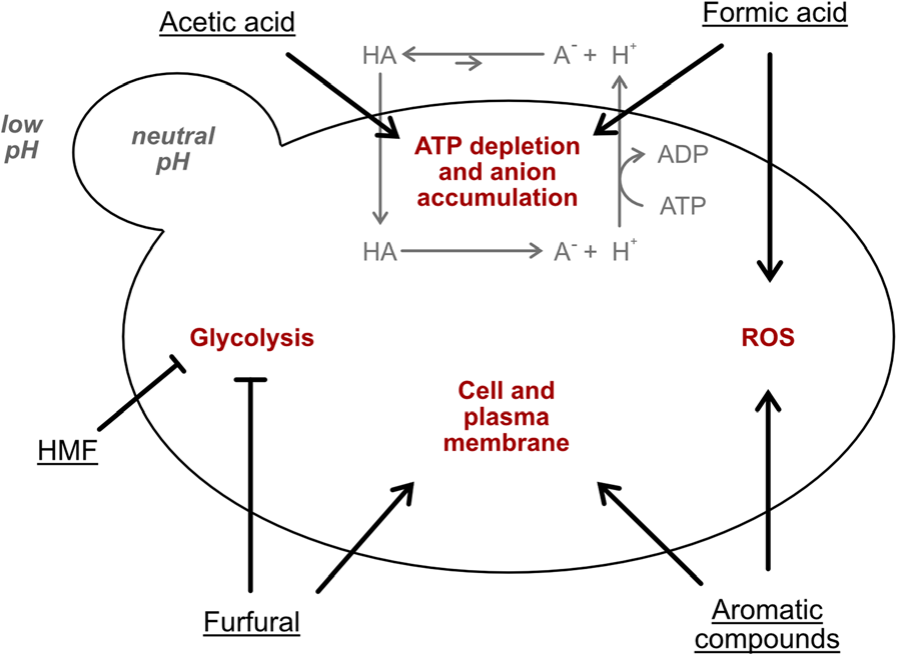
Mechanisms of different inhibitors commonly found in a hydrolysate. Weak acids (e.g. acetic and formic acid) diffuse into the cell and release protons. To maintain a neutral pH, cells transport the protons out under the consumption of ATP. HMF and Furfural inhibit glycolysis which leads to a slower growth rate. Furfural and aromatic compounds damage both the cell and plasma membranes. Formic acid and aromatic compounds cause the formation of reactive oxygen species (ROS). Adapted from [12]

The most common weak acids present in lignocellulosic hydrolysates are acetic acid and formic acid [12]. Acetic acid is formed by deacetylation of hemicelluloses and formic acid is a product of HMF and furfural breakdown [13, 14]. The inhibitory effect of weak acids is thought to be mainly associated with the uncoupling phenomena [15]. The undissociated form of a weak acid diffuses across the plasma membrane and dissociates due to a higher intracellular pH, decreasing the cytosolic pH (Fig. 1). To counter the lowering of intracellular pH, protons are pumped out causing ATP depletion and anion accumulation, which impact cell viability and reduces biomass formation [16].

Aromatic compounds are released during the process of acid hydrolyzation of carbohydrates and lignin. Depending on the type of biomass, lignin has a different degree of methoxylation, internal bonding and association with cellulose and hemicellulose. This results in a wide range of different types of aromatic compounds, such as cinnamic acid and coniferyl aldehyde (4-hydroxy-3-methoxycinnamaldehyde) [12, 17]. The inhibitory effects of cinnamic acid can be attributed to the weak acidity of the molecule and the hydrophobicity of cinnamic acid also affects the integrity of biological membranes by inducing a non-specific increase of membrane permeability [18, 19].

Most of the information about the inhibitory compounds in lignocellulosic hydrolysates are derived from studies on *Saccharomyces cerevisiae* [20]. Although it has been shown that hydrolysate can be used to cultivate *Y. lipolytica* [21], the individual effects of these compounds on *Y. lipolytica* are much less studied. To understand the role of these inhibitors in *Y. lipolytica*, we characterized its tolerance to individual representative compounds from the main classes of inhibitors found in lignocellulosic hydrolysates (acetic acid, formic acid, furfural, HMF, cinnamic acid, and coniferyl aldehyde) and the combination of these. Our study gives insight into the inhibitor tolerance of *Y. lipolytica* and serves to enable the targeted development of resistant and robust *Y. lipolytica* strains.

## Results and discussion

The overall goal of our studies is to enable the use of carbon sources present in hydrolysates of lignocellulosic biomass for triacylglycerides (TAG) production. Here we have focused on understanding the individual and synergetic effect of inhibitors found in lignocellulosic hydrolysates on the growth.

### Construction and characterization of a xylose consuming strain (SZYL004).

The efficient utilization of lignocellulosic hydrolysate requires that xylose can be used as a carbon source. Therefore, we genetically engineered *Y. lipolytica* to improve xylose catabolism. The genome of *Y. lipolytica* contains open reading frames for all three proteins required to catabolize xylose: xylose reductase (XR; YALI0D07634/YALI1_D09870g), xylitol dehydrogenase (XDH; YALI0E12463/YALI1_E15452g), and xylulose kinase (XK; YALI0F10923/YALI1_F14583g) [22]. These genes are homologous to those of yeast species able to metabolize xylose by a pathway in which, after uptake, D-xylose is first reduced to xylitol by XR and further oxidized to D-xylulose mediated by XDH. In a final step, D-xylulose is then phosphorylated by XK to yield D-xylulose-5-P which can enter the pentose phosphate pathway [7]. However, many strains of *Y. lipolytica* do not grow on xylose, or their growth is highly depended on culturing conditions and pre-adaptation [7].

**Fig. 2:**
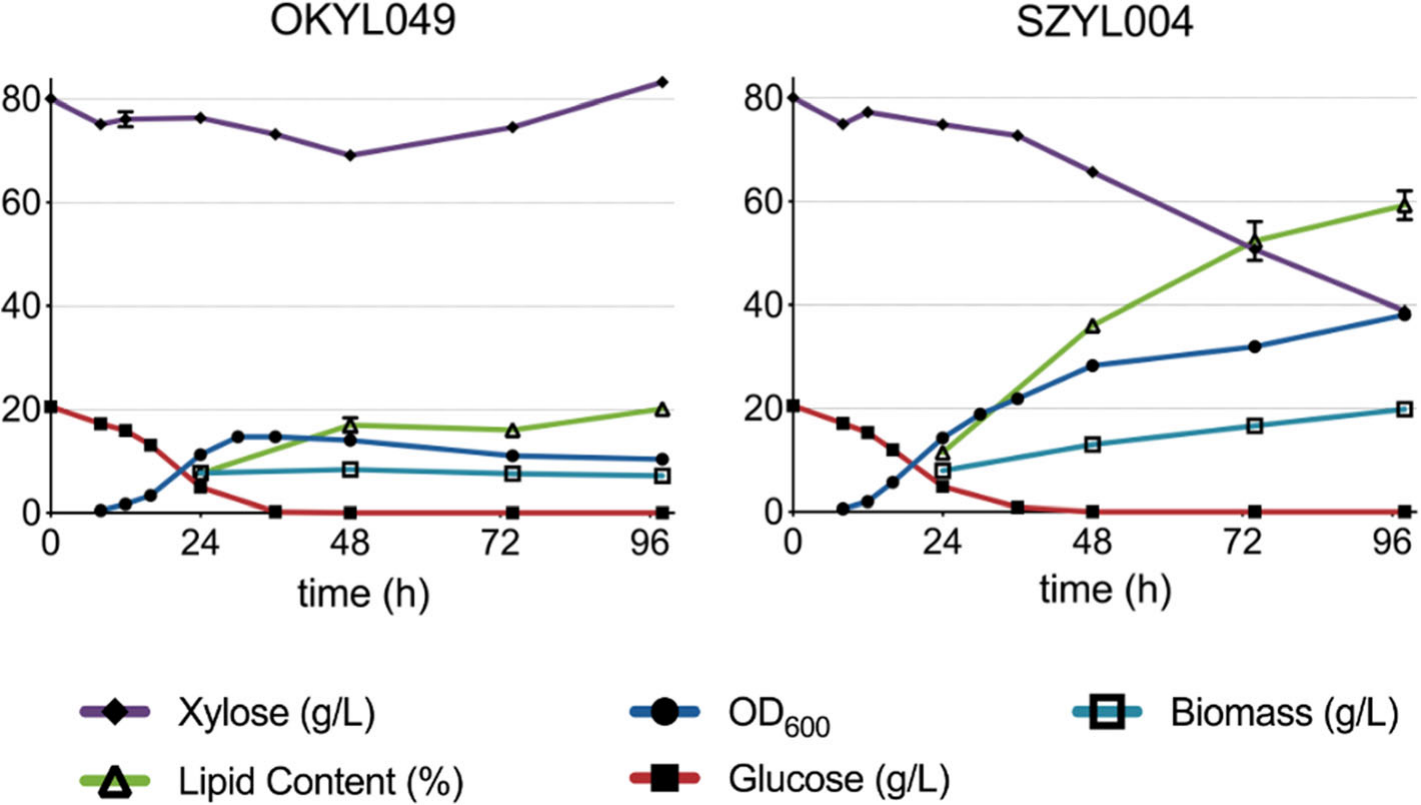
Growth performance of SZYL004 in shake flask with different carbon sources. Shake flask cultivation of OKYL049 and SZYL004 on LPU + 20 g/L glucose + 80 g/L xylose. All curves represent the average, and error bars the standard deviation of triplicates, respectively

We constructed a xylose consuming strain (SZYL004) by overexpressing the three native xylose genes XR, XK, and XDH, under the control of pPYK1, pTEF1, and pGAPDH promoters respectively. An overexpression of only XK and XDH resulted in only minor growth on xylose (Figure S1), suggesting that the native expression level of XR is not sufficient which is in contrast to previous reports [23, 24]. The xylose consuming strain (SZYL004) showed an increased lag phase when cultivated in media containing only xylose as a carbon source (Figure S2), likely because of an insufficient transport of xylose into the cell, which could be further improved by overexpressing xylose transporter proteins [25]. This lag phase could be prevented using media containing both glucose (2%) and xylose (8%). In this media, SZYL004 did show an increased lipid production (up to 60%) and increased biomass production compared to its parental strain OKYL049 (Fig. 2).

### The tolerance of Y. lipolytica to individual inhibitors

To determine which inhibitor classes pose a problem for *Y. lipolytica*, we decided to evaluate the effect of a set of individual representative inhibitory compounds (chosen from [26]). First, we tested the effect of six single inhibitors in different carbon sources to assess the tolerance range of *Y. lipolytica*. Then the inhibitors were combined to mimic the composition of a real hydrolysate [26].

### Effect of the overexpression of the xylose genes on the inhibitor tolerance

**Fig. 3:**
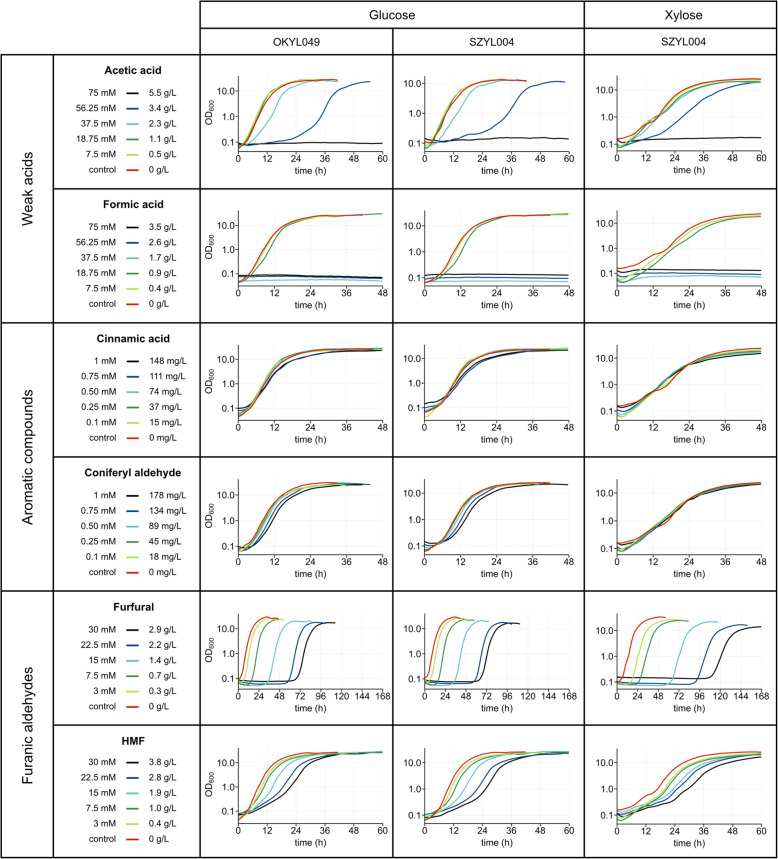
Growth curves of *Y. lipolytica* strains with different inhibitors. Strains were cultured in 96-well plates and the OD_600_ was measured with the growth profiler every 30 min. Media contained 100 g/L sugar. The curves represent the average of triplicates

Inhibitor tolerance in the xylose engineered strain (SZYL004) and its parental strain (OKYL049) were evaluated to exclude that the overexpression of the three native xylose genes (XR XK, and XDH) affect inhibitor tolerance, e.g. by affecting the central metabolism. This was investigated by comparing the inhibitor effects on the xylose engineered strain SZYL004 and its parental strain OKYL049 during growth on glucose. Both strains showed a very similar growth behavior in all tested inhibitors (Fig. 3), indicating that the overexpression of the three native xylose genes does not significantly affect inhibitor tolerance of Y. *lipolytica*.

### Effect of the carbon source on the inhibitor tolerance

Xylose catabolism affects tolerance to furfural, coniferyl aldehyde and acetic acid, but not to HMF, formic acid, and cinnamic acid (Fig. 3). It is possible that differences in metabolism caused by the choice of carbon source could affect inhibitor tolerance. The strain growing on xylose showed a lower growth rate and a longer lag phase than the strain growing on glucose. However, no major carbon source dependent differences in inhibitor tolerance were observed in the presence of either formic acid, cinnamic acid and HMF (Fig. 3).

However, when SZYL004 was growing in the presence of furfural with glucose or xylose as carbon and energy source a clear difference was observed (Fig. 3). At the same furfural concentration, SZYL004 growing on xylose displays a longer lag phase than during growth on glucose. For example, at 30 mM furfural SZYL004 on glucose grows after 70 h, while on xylose only grows after 115 h. It is likely that the introduction of the two genes XR and XD lead to cofactor imbalance compared to growth on glucose, since XR uses NADPH and XDH uses NAD^+^ as a cofactor [24]. Moreover, *Y. lipolytica* cannot convert cytosolic excess of NADH into NADPH [27]. Additionally, furfural detoxification causes a decrease in the intracellular levels of NADPH in *S. cerevisiae* [10]. The combination of these two factors increases the burden on the cell while metabolizing xylose, which could explain the higher resistance to furfural of SZYL004 on glucose than on xylose.

For coniferyl aldehyde, we saw a tendency to less sensitivity during growth on xylose compared to glucose. In glucose, there was a small increase in lag phase up to concentrations of 1 mM, which was not observed during growth on xylose (Fig. 3).

A lower sensitivity of xylose grown cells was also observed for acetic acid. While in glucose a concentration of 37.5 mM acetic acid increased the lag phase, this was not observed on xylose (Fig. 3).

### Effect of weak acids on the growth of Y. lipolytica

Acetic acid was well tolerated by *Y. lipolytica* up to 18.75 mM. At concentrations between 18.75 mM and 56.25 mM, both the lag phase and growth rate were affected. Acetic acid did not allow growth at concentrations of 75 mM (Fig. 3).

**Fig. 4:**
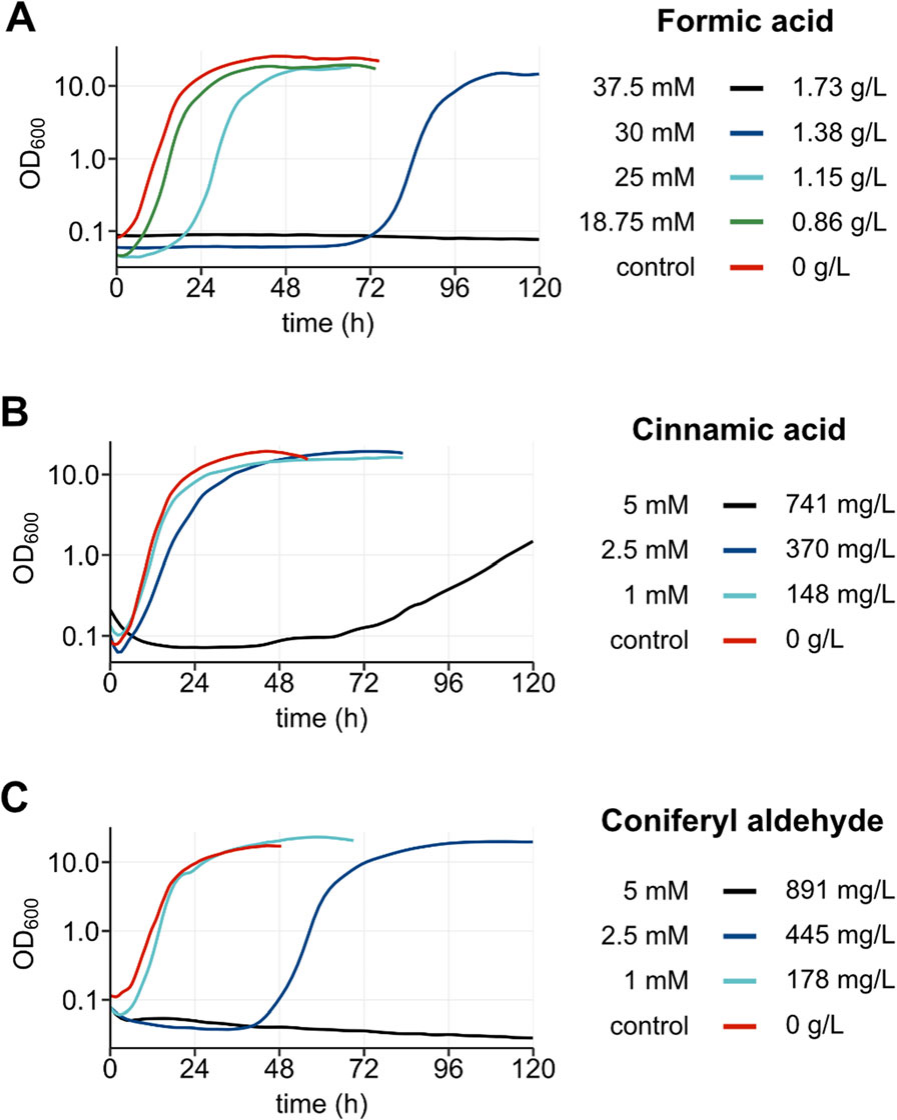
Extended analysis of the inhibition by aromatic compounds and formic acid. Growth curves of SZYL004 in LPU + 100 g/L xylose and different inhibitors. Cells were cultured in 96-well plates and the OD_600_ was measured with the growth profiler every 30 min. The curves represent the average of triplicates. **a**: Growth with formic acid, **b**: cinnamic acid, and **c**: coniferyl aldehyde

Formic acid showed similar effects as acetic acid, but the tolerance range was narrower. At concentrations up to 18.75 mM formic acid did not affect the growth of *Y. lipolytica*. Formic acid did not allow growth at concentrations of 37.5 mM and higher (Fig. 3). To further characterise the formic acid sensitivity, intermediate concentrations were tested in xylose containing media. The results show that growth was possible up to a concentration of 30 mM formic acid. However, there was a major increase in the lag phase (25 mM: 18 h; 30 mM: 72 h) (Fig. 4 a). This shows that there is a threshold for formic acid tolerance in our *Y. lipolytica* strain of approximately 25 mM.

Acetic acid and formic acid are toxic compounds because they lead to intracellular anion accumulation. The undissociated form of the acid can diffuse throught the plasma membrane and dissociate inside the cell, leading to anion accumulation. Formic acid shows higher toxicity than acetic acid likely due to a lower pKa value (3.75 at 25 °C) than acetic acid (4.75 at 25 °C). Therefore, at the same molarity, the intracellular pH will be more affected by formic acid than by acetic acid [12]. Formic acid also has a smaller molecular size than acetic acid and thus can diffuse more easily through the plasma membrane [12]. Formic acid has been shown to induce ROS production and to induce apoptosis in *S. cerevisiae* [28]; a similar effect might contribute to the formic acid toxicity in *Y. lipolytica*. Furthermore, acetic acid could be less toxic than formic acid because the dissociated form (acetate) can be activated to acetyl-CoA in the cytoplasm by acetyl-CoA synthetase and then be incorporated into lipids or transported into the mitochondria to enter the tricarboxylic acid (TCA) cycle [29].

The tolerance of another oleaginous yeast, *Rhodosporidium fluviale,* was previously tested and it was found that its growth was strongly affected at a concentration of 10.9 mM formic acid and 16.7 mM of acetic acid [14]. Thus, *Y. lipolytica* seems to display an advantage over both *S. cerevisiae* and *R. fluviale* in this respect.

### Effect of aromatic compounds on the growth of Y. lipolytica

Cinnamic acid shows strong effects on the growth at concentrations above 2.5 mM, while coniferyl aldehyde has strong effects at 2.5 mM.

The inhibitory effects of cinnamic acid on *Y. lipolytica* was initially tested for concentrations up to 1 mM (148 mg/L) (Fig. 3). In that range, cinnamic acid did not show any effect on the growth parameters. As a follow up we also tested cinnamic acid concentration of 2.5 mM and 5 mM to find the upper limit of cinnamic acid tolerance for *Y. lipolytica* (Fig. 4 b). *Y. lipolytica* displayed only a slight reduction in growth rate and yield in 2.5 mM cinnamic acid (370 mg/L). However, at concentrations of 5 mM, the overall growth was strongly affected.

The high tolerance towards cinnamic acid is not only interesting in the context of hydrolysate utilization, but also for other metabolic engineering applications. For instance, cinnamic acid can be converted to p-coumaric acid, which is an intermediate in the production of flavonoids, e.g. kaempferol or naringenin [30]. Previous studies on *S. cerevisiae* suggest that increasing concentrations of cinnamic acid up to 35 mg/L led to an increase of the lag phase and a gradual reduction of the growth rate [31]. Even though with engineering efforts *S. cerevisiae* tolerance to cinnamic acid can be improved [18], the high cinnamic acid tolerance of *Y. lipolytica* could allow high production of flavonoids in the future with less engineering efforts.

The inhibitory effects of coniferyl aldehyde on *Y. lipolytica* were at first also tested for concentrations only up to 1 mM (178 mg/L). These concentrations had a minor impact on the growth on glucose, but not on xylose-based media (Fig. 3**)**. To explore the limit of coniferyl aldehyde tolerance, concentrations of 2.5 mM (445 mg/L) and 5 mM (891 mg/L) were tested. In 2.5 mM *Y. lipolytica* showed a prolonged lag phase, but a similar growth rate and maximum OD_600_ compared to that of the control (Fig. 4 c). Growth was not detectable at coniferyl aldehyde concentrations of 5 mM even after 120 h of cultivation. The mechanism of coniferyl aldehyde toxicity has not been fully elucidated, but a correlation with ROS accumulation has been observed. A transcriptomic analysis of a strain of *S. cerevisiae* evolved for tolerance to CA showed an upregulation of 11 genes with a role in oxidative stress response [32].

### Effect of furanic aldehydes on the growth of Y. lipolytica

**Fig. 5:**
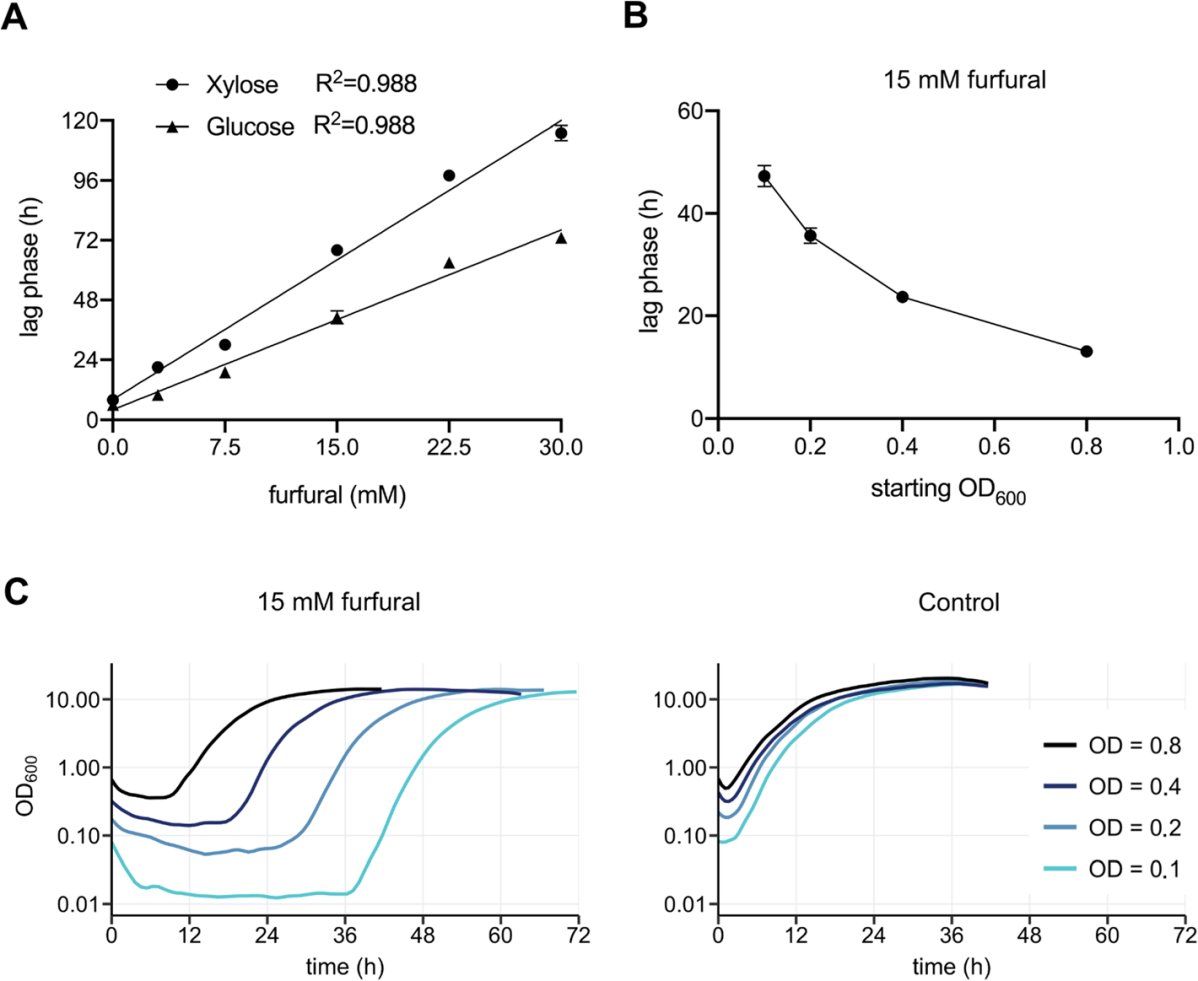
Investigation of furfurals effect on the lag phase. **a**: linear regression of lag phase vs. furfural concentration for xylose and glucose. **b**: plot of the lag phase length vs. different starting OD_600_. **c**: Impact of inoculum size on lag phase in LPU + 100 g/L xylose with 15 mM furfural. SZYL004 was inoculated to different starting OD_600_ and was cultured in 96-well plates. The OD_600_ was measured with the growth profiler every 30 min. The curves represent the average of triplicates

Furfural increases the lag phase in a linear matter, while HMF reduces the growth rate. To investigate the effect of furfural, our strains were cultivated in media containing different concentrations of furfural (Fig. 3) and we found that every 1 mM of furfural increased the lag phase of SZYL004 by 2.4 h and 3.7 h with glucose or xylose as a carbon source, respectively (Fig. 5 a). *Y. lipolytica* cannot metabolize furfural or HMF to enter cellular metabolism, but has non-specific enzymes such as reductases and dehydrogenases that can reduce or oxidase these compounds to less inhibitory alcohols (furfuryl alcohol and HMF alcohol) or acids (furoic acid and HMF acid) [33, 34]. After inhibitor degradation, cells show normal growth. Increasing the inoculum density yielded faster detoxification and a shorter lag phase (Fig. 5 b and c) and can be considered one way to handle the inhibitory effects of furfural in hydrolysates, as previously suggested [35].

The addition of HMF led to a complex phenotype regarding the growth parameters (Fig. 3). With increasing HMF concentration we observe two different growth phases. While an increase of lag phase caused by furfural corresponded to a lack of all growth, in HMF we observe a low growth rate until an OD_600_ of approximately 0.3, at which point the growth rate increased up to a rate similar to the control. The most straightforward explanation for this behavior is that HMF only partially inhibits the growth until the HMF is detoxified, after which normal growth resumes.

Previous studies testing the tolerance of *Y. lipolytica* found no tolerance to furfural when testing its growth on 0.5 g/L furfural (5 mL media in test tubes at 23 °C) [36]. Our strain showed an extended lag phase but grew in furfural concentrations up to 2.9 g/L. These results indicate that there are differences in tolerance between different strain isolates.

### Effect of an inhibitor mixture on the growth of Y. lipolytica

**Fig. 6:**
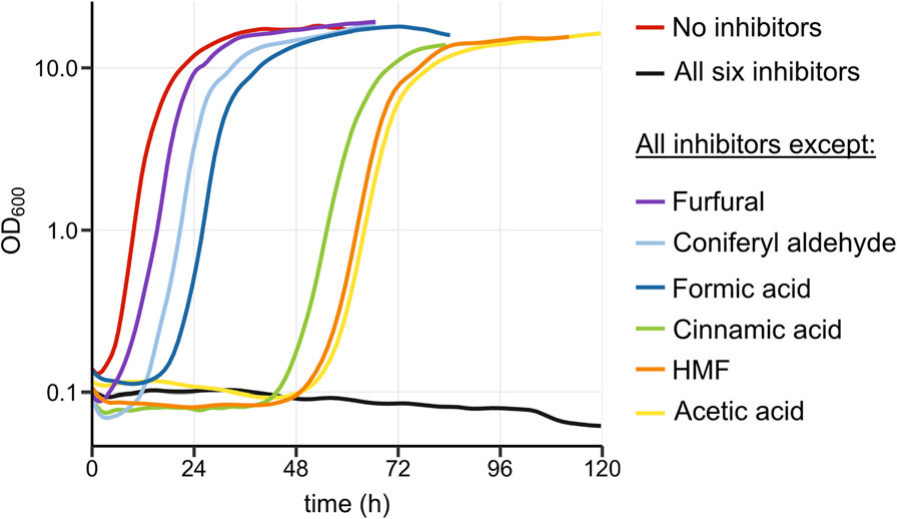
Growth curves in different inhibitor mixtures. SZYL004 was cultured LPU + 100 g/L xylose in 96-well plates and the OD_600_ was measured with the growth profiler every 30 min. The curves represent the average of quadruplicates. Inhibitor concentrations used: 15 mM acetic acid, 15 mM formic acid, 0.2 mM cinnamic acid, 0.2 mM coniferyl aldehyde, 6 mM furfural, and 6 mM HMF

During the process of hydrolysate treatment, multiple inhibitors are formed, which affect the growth performance of microorganisms in a synergistic manner [8]. *Y. lipolytica* SZYL004 was tested during growth on xylose based media, supplemented with an inhibitor mixture mimicking the composition of a real hydrolysate [26](Fig. 6). The concentrations of the inhibitors correspond to 20 percentage of the maximum concentrations that were initially tested in previous experiments. The strain did not grow in the full inhibitor mixture. We therefore decided to evaluate the contribution of the individual inhibitory compounds in causing the growth phenotype. To this end, we evaluated growth in mixtures containing only five of the six inhibitors, leaving out one inhibitor at a time. We observed that omission of any of the six different inhibitors could partially rescue the growth (Fig. 6). The omission of furfural showed the strongest impact on the growth, followed by coniferyl aldehyde, and formic acid. These results are not surprising for furfural and formic acid since in previous experiments with single inhibitory compounds both of them showed a strong impact on cell growth (Fig. 3). However, that coniferyl aldehyde omission resulted in such a strong recovery of growth was highly surprising, since we observed high tolerance (up to 2.5 mM) in previous experiments with single inhibitors (Fig. 4). This implies that inhibitor toxicity is highly dependent on synergistic effects. Phenolic compounds (such as coniferyl aldehyde) [20], furfural [9], and formic acid [28] have all been reported to induce ROS formation. Therefore, it is likely that the synergistic effects we observe could be linked to ROS-related effects. While the amount of ROS generated by each inhibitor might be tolerated, combining multiple inhibitors could put a burden on the cell that leads to growth arrest.

## Conclusions

Our work shows the tolerance of *Y. lipolytica* to different inhibitors commonly found in lignocellulosic hydrolysates. Comparing growth on glucose and xylose we only found minor carbon source dependent effects on inhibitor tolerance. Furfural and formic acid were identified as the major growth inhibitory compounds, both individually or in combination with other inhibitors/compounds. We showed that the furfural mediated effect on the lag-phase can be overcome by increasing the culture inoculum. We also find strong evidence for synergistic effects of the inhibitory compounds, especially when coniferyl aldehyde is present in the mixture.

Future research should focus on tolerance of formic acid and aromatic aldehydes. A possible engineering approach could therefore be the overexpression of inhibitor degrading enzymes such as formate dehydrogenases or transporters, or optimization of the culturing conditions. Our study provides data on the effects of individual inhibitory compounds, as well as data on synergistic effects. It is our belief that this will serve as a basis for design of experiments directed toward increasing tolerance of *Y. lipolytica*. Thereby, enabling the use of *Y. lipolytica* for hydrolysate based bioprocesses.

## Material and methods

### Strains and strain construction

All strains in this study are derived from the *Yarrowia lipolytica* strain ST6512 [37]. ST6512 was derived from the W29 background strain (Y-63746 from the ARS Culture Collection, Peoria, USA; a.k.a. ATCC20460/CBS7504) which has been engineered to harbor a KU70::Cas9-DsdA to allow fast marker-free genomic engineering using the EasyCloneYALI toolbox [38].

Transformation of *Y. lipolytica* was performed using a lithium-acetate based heat shock method previously described [39]. Briefly, the strain was plated on YPD agar plates and grown at 30 °C for 18 h. The cells were washed off the plate and washed twice with water, after which a pellet of 3 OD_600_ units in 1 mL, corresponding to roughly 1.6 × 10^7^ cells, was used for each transformation.

For gene deletions, 3 μL of a deletion fragment was used: deletion fragments were constructed from equal amounts of two single-stranded oligonucleotides (around 100 bp; 100 pmol/μL; sequence in supplementary) which were incubated for 5 min at 95 °C and allowed to cool down to room temperature. For integration 1 μg of repair fragment was used.

The cells were carefully resuspended in transformation mix (sterile PEG (43.8% v/v), lithium acetate (0.1 M), boiled single-stranded DNA from salmon testes (0.25 mg/mL) and sterile dithiothreitol (DTT) (100 mM)) together with repair fragment DNA and 500 ng gRNA plasmid, and incubated at 39 °C for 1 h. The cells were spun down, resuspended in YPD media and incubated at 30 °C at 200 rpm for 2 h. Afterwards, cells were spun down and resuspended in water before plating on agar plates containing 250 mg/L nourseothricin. After 3–4 days of incubation at 30 °C colonies could be screened via colony PCR.

OKYL049 is an obese strain in which overexpression of *DGA1* was first introduced, followed by deletion of *ARE1* to increase TAG accumulation and abolish sterol ester formation. *MHY1* was deleted to prevent hyphae formation [40]. The strains SZYL002 (overexpressing XK and XDH) and SZYL004 (overexpressing XK, XDH, and XR) are derived from OKYL049 (Table 1). The DNA-constructs and primers used are detailed in the supplementary file.

**Table 1:** Strains used in this study and their genotypes

Name	Genotype description	Reference
**ST6512**	W29 + MATa ∆ku70::Cas9::DsdA	[37]
(aka wild type)		
**OKYL049**	ST6512 + E1::pTef1in + DGA1 + tPEX20 Δare1 Δmhy1	this paper
(aka *obese strain)*		
**SZYL002**	OKYL049 + C3::[(pTef1 + XK + tPex20)-(pGAPDH+XDH + tLip2)]	this paper
**SZYL004**	OKYL049 + C3::[(pTef1 + XK + tPex20)-(pGAPDH+XDH + tLip2)-(pPYK1 + XR + tPex16)]	this paper

### Media and growth conditions

Lipid production media (LPU) consisted of 1.5 g/L yeast extract, 0.85 g/L, casamino acids, 1.7 g/L Yeast Nitrogen Base without amino acids and ammonium sulfate, 5.1 g/L potassium hydrogen phthalate buffer adjusted to pH 5.5, 100 g/L glucose, and either 0.5 g/L urea [41].

YPD plates contained 20 g/L peptone from meat, 10 g/L yeast extract, 20 g/L glucose, and 20 g/L agar. For selection YPD plates were supplemented with 250 mg/L Nourseothricin. LB plates contained 10 g/L peptone from casein, 10 g/L NaCl, 5 g/L yeast extract, 16 g/L agar, and were set to pH 7.0 with 5 M NaOH.

For shake flask experiments precultures were grown in 8 mL LPU media containing the same carbon source as the main culture (glucose or xylose). Precultures were harvested at OD_600_ between 5 and 7, and 25 mL media were inoculated at OD_600_ 0.1 and cultivated at 30 °C in 250 mL Erlenmeyer flasks at 200 rpm shaking. Each experiment was carried out in triplicate.

Inhibitors were diluted from concentrated solutions to the concentrations indicated in the figures.

*Escherichia coli* DH5alfa was used for plasmid construction and purification and was cultivated in LB broth or on agar plates supplemented with 100 μg/mL ampicillin at 37 °C.

### Growth profiler

*Y. lipolytica strains* OKYL049, SZYL002 and SZYL004 were cultivated in 96-well plates, at 30 °C and 200 rpm, and growth performances were determined with Growth Profiler 960 (Enzyscreen B.V., Heemstede, The Netherlands) with OD_600_ measurement every 30 min. We have found in shake flask experiments (Figure S2) that OD and biomass correlate well. To determine the growth performances, 150 μl of LPU media were supplemented with six types of growth inhibitors [26] in different dilutions. Precultures were grown in inhibitor-free LPU media containing the same carbon source as the main culture (glucose or xylose) and were harvested at OD_600_ between 5 and 7 to inoculate to a starting OD_600_ of 0.1. Each experiment was carried out in triplicate. For the sake of figure clarity, growth curves were cropped after reaching stationary phase, and error bars are not displayed. Growth curves were smoothed using the LOESS regression method via the geom_smooth function from the R package ggplot2 (geom_smooth(method = LOESS, span = 0.1, se = FALSE, size = 1.5)).

From the obtained curves the lag phase length, the growth rate and the yield (= the final OD_600_) were determined. The lag phase length was defined as the time at which the OD_600_ exceeded 0.25. The growth rate was calculated using the R package “growthrates” [42]. The yield was defined as the OD_600_ reached by the culture at the end of the fermentation.

### Lipid extraction and quantification

*Y. lipolytica* strains were cultivated in LPU media and samples were taken after 24, 48, 72 and 96 h for fatty acid methyl ester (FAME) extraction to measure cellular lipid content. The protocol used was previously described [40, 43]. In short, 100 μL of cell culture was spun down, the supernatant was discarded, and the cells were washed twice with 1 mL water. The suspension was spun down again and the supernatant was removed. The cell pellet was dried in a vacuum dry freezer for 1 day. Then 40 μg of triheptadecanoin (TAG(17:0/17:0/17:0)) was added to the cell pellet as the internal standard. 500 μL of methanol solution containing 1 M NaOH was added and the samples were vortexed at 1200 rpm at room temperature for 1 h. The solution was neutralized by carefully adding 80 μL of 50% sulfuric acid. The FAMEs were extracted by adding 500 μL hexane. Phases were separated by centrifugation for 1 min at 10.000 rcf. 200 μL of the upper hexane phase was mixed with 800 μL hexane and 1 μL of this sample was analyzed on GC-MS (Thermo Scientific Trace 1310 coupled to a Thermo Scientific ISQ LT with a ZBFAME column (Phenomenex, length: 20 m; Inner Diameter: 0,18 mm; Film Thickness: 0,15 μm)).

To allow a calculation of lipid content per cell dry weight, the dry weight of each culture was calculated as follows: 1 mL of culture was spun down and washed twice. The final suspension was then filtered, after which the filter was washed with 10 mL of milliQ water before drying and weighing.

### High-performance liquid chromatography

To quantify extracellular metabolites, fermentation samples were prepared by taking 1 mL of culture, centrifuging for 5 min at 3000 rcf, and using the supernatant for high-performance liquid chromatography (HPLC) analysis. The HPLC system UltiMate® 3000 (Dionex) was utilized with an Aminex® HPX-87H ion exclusion column (Bio-Rad). 5 mM H_2_SO_4_ was used as eluent at a flow rate of 0.6 mL/min. Glucose and xylose were quantified using a refractive index detector (Shodex ri-101).

## Supplementary Information

**Additional file 1.**

**Additional file 2.**

**Additional file 3.**

## Data Availability

All the raw data analysed during this study are included in the supplementary information. “Data Figure 2.xlsx” includes the data analyzed to prepare figure 2. “Data Figure 3.xlsx” includes the data analyzed to prepare figure 3. “Data Figure 4.xlsx” includes the data analyzed to prepare figure 4. “Data Figure 5.xlsx” includes the data analyzed to prepare figure 5. “Data Figure 6.xlsx” includes the data analyzed to prepare figure 6. “Data Supplementary Figure 1.xlsx” includes the data analyzed to prepare supplementary figure 1. “Data Supplementary Figure 2.xlsx” includes the data analyzed to prepare supplementary figure 2.

## Abbreviations

Coniferyl aldehyde: 4-hydroxy-3-methoxycinnamaldehyde
FAME: Fatty acid methyl ester
Furfural: 2-furaldehyde
GC-MS: Gas chromatography-mass spectrometry
HMF: 5-hydroxymethylfurfural
HPLC: High performance liquid chromatography
LPU: Lipid production media
ROS: Reactive oxigen species
TAG: Triacylglyceride
TCA: Tricarboxylic acid cycle
XDH: Xylitol dehydrogenase
XK: Xylulose kinase
XR: Xylose reductase
