# Supplementary material for: Tolerance of Yarrowia lipolytica to inhibitors commonly found in lignocellulosic hydrolysates
Source: BMC Microbiol. 2021 Mar 8;21:77. doi: 10.1186/s12866-021-02126-0 (PMC7938539; doi:10.1186/s12866-021-02126-0)
Supplement: Supplementary file 3 — Additional file 3. [file 12866_2021_2126_MOESM3_ESM.pdf]

<u>Supplementary</u>

Tolerance of *Yarrowia lipolytica* to inhibitors commonly found in lignocellulosic hydrolysates

Oliver Konzock[1,*], Simone Zaghen[1,*], Joakim Norbeck[1#]

1 Chalmers University of Technology, Department of Biology and Biological Engineering, division of Systems and Synthetic Biology, Göteborg, Sweden

* these authors contributed equally to this work

# Corresponding author: norbeck@chalmers.se

Content

- Supplementary figures
- List of plasmids
- List of primers
- List of sequences

Supplementary figures

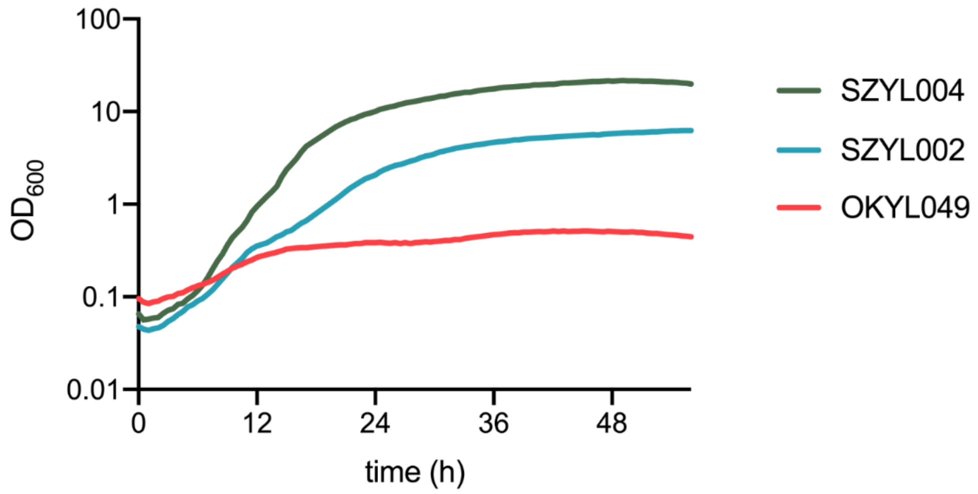

Figure S1: Growth curves of *Y. lipolytica* strains expressing xylose genes. Strains were precultured for 18 hours in LPU media with 10% glucose and then grown in LPU media with 10% xylose as sole-carbon source Cells were cultured in 96-well plates and the OD_{600} was measured with the growth profiler every 30 minutes. The curves represent the average of triplicates.

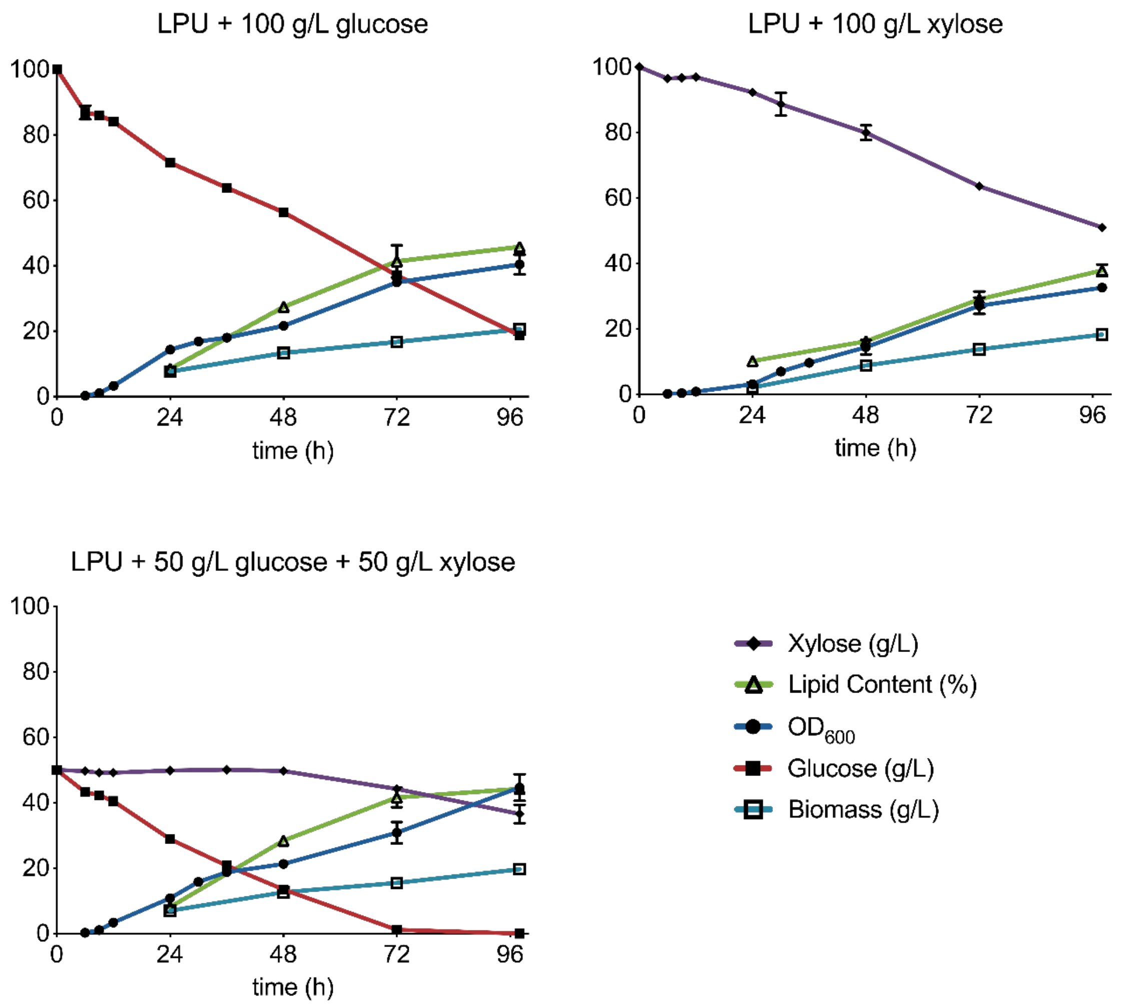

Figure S2: Shake flask cultivation of SZYL004 on different carbon sources. Glucose, xylose, OD_{600}, biomass and lipid content curves of SZYL004 on LPU containing different percentages of glucose and xylose as carbon sources. Strains were cultured in shake flasks for 96 hours. The curves represent the average, and error bars the standard deviation of triplicates, respectively.

Supplementary list 1: List of plasmids used in the study - Konzock, Zaghen, Norbeck 2021

Name	Plasmid contains	Yarrowia marker	Purpose of plasmid	Reference
**pCfB6630**	gRNA C3 locus	nourseothricin	expresses gRNA for cut in C3 locus of YaliEASYCLONE kit	EasyCloneYALI: CRISPR/Cas9-Based Synthetic Toolbox for Engineering of the Yeast Yarrowia lipolytica. Holkenbrink C, Dam MI, Kildegaard KR, Beder J, Dahlin J,
**pCfB6633**	gRNA E1 locus	nourseothricin	expresses gRNA for cut in E1 locus of YaliEASYCLONE kit	
**SZ_EC_01**	(pTef1+XK+tPex20)-(pGAPDH+XDH+tLip2) in Int_C3	marker-free	cut with NotI to linearize repair fragment for C3 locus, overexpressing XK, and XDH	this paper
**SZ_EC_02**	(pTef1+XK+tPex20)-(pGAPDH+XDH+tLip2)-(pPYK1+XR+tPex16) in Int_C3	marker-free	cut with NotI to linearize repair fragment for C3 locus, overexpressing XK, XDH, and XR	this paper, available on Addgene
**OKEC012**	pTef1in+DGA1+tPEX20 in Int_E1	marker-free	cut with NotI to linearize repairfragment for E1 locus, overexpressing DGA1	this paper

Supplementary list 2: List of primer sequences used in the study - Konzock, Zaghen, Norbeck 2021

	Name	Sequence	Target
**repair fragments for deletion**	are1_deletion-box-1	ggggcaaaaaggatacaagtcgttcttacaagacactctccgcacacacagcacagtcgcttcaccacttgtgcctatagaatgtcaaccactgtcaac	fused together to form repair fragment for ARE1 deletion
	are1_deletion-box-2	gttgacagtggttgacattctataggcacaagtggtgaagcgactgtgctgtgtgtgcggagagtgtcttgtaagaacgacttgtatcctttttgcccc	
	MHY1_deletion_box1	cgccaaaaccatcccaatcgaaaaacaaaaagtcatagtacattatcgccaaaaggtagagcgccttctagtctccgctccatttttttattgtaaccag	fused together to form repair fragment for MHY1 deletion
	MHY1_deletion_box2	ctggttacaataaaaaaatggagcggagactagaaggcgctctaccttttggcgataatgtactatgactttttgtttttcgattgggatggttttggcg	
**cut sites to build gRNA plasmids** *	ARE1_Cut-site_FW	gaagagctcaatgtgcccgagttttagagct	cut sites for ARE1
	ARE1_Cut-Site_RV	tcgggcacattgagctcttctaaccaacct	
	ARE1_Cut-site_2_FW	gttttctgcagcatggccaggttttagagct	
	ARE1_Cut-Site_2_RV	ctggccatgctgcagaaaactaaccaacct	
	MHY1_cut-site_1_FW	aggcgacagcatgtaaatgggttttagagct	cut sites for MHY1
	MHY1_cut-site_1_RV	ccatttacatgctgtcgccttaaccaacct	
	MHY1_cut-site_2_FW	ctcgagaggcgagtaagtgggttttagagct	
	MHY1_cut-site_2_RV	ccacttactcgcctctcgagtaaccaacct	
**screening primer for deletions**	are1-del_FW_Screen	tgttgctgggtaagtcaaacc	ARE1 deletion screening: 616 bp for deletion / 2248 bp for WT
	are1-del_RV_Screen	aacttcagtggaagcattgtagc	
	MHY1-del_FW_Screen	tctttcaaccgtcccacacc	MHY1 deletion screening: 675 bp deletion / 1533 bp MHY1 WT
	MHY1-del_RV_Screen	caaagtggacaagcgactgc	
**primer to build plasmid with xylose pathway (SZ_EC_02)**	XK_Gibson_FW	ACACTTCAACGGAATGCGTGCGATttatttctccaggcaggcg	Primers with overhangs used to create the fragments for the gibson assembly of SZ_EC_02
	XK_Gibson_RV	agaatcattcaaaatgtatctcggactggatctttcg	
	Tef1_Gibson_FW	gtccgagatacattttgaatgattcttatactcagaaggaaatgc	
	Tef1_Gibson_RV	atttcaaccgcgatcgcagagaccgggttggcggcg	
	GAPDH_Gibson_FW	ccggtctctgcgatcgcggttgaaatgaatcggccg	
	GAPDH_Gibson_RV	ggttagaagacattgttgatgtgtgtttaattc	
	XDH_Gibson_FW	acacacatcaacaatgtcttctaacccgtcatttg	
	XDH_Gibson_RV	CCGAACAGAAGGAATGCACGCGTActactcctcctcgggaccgtc	
	pPYK1_Gibson_FW	gcttgaaggacattgtaactgtggtgtgaatttctcc	
	pPYK1_Gibson_RV	gccgattcatttcaaccGCGattgcgcgcttctgttgtttgc	
	XR_Gibson_FW	aatcatcaatcgcttaggcgaaaatgggaaggttag	
	XR_Gibson_RV	caccacagttacaatgtccttcaagctcgcctc	

	pex16_Gibson_FW	cgccgccaacccggtctctgcgataagcttcgagaagcccgaac	
	pex16_Gibson_RV	cattttcgcctaagcgattgatgattggaaacac	
**screening primers for integration**	PR-14838	aaacctgtcgtatgagctac	screening of C3 locus: positive integration: RP-14838 + RP-8859 = 915 bp; negative: RP-14838 + RP-14588 = 1106 bp
	PR-8859	aagtgtggatggggaagtgag	
	PR-14588	acgcgauaattattgcacaggacacac	
	PR-14442	agttgtgaccaagacaaatg	screening of E1 locus: positive integration RP-14442 + RP-8859 = 891 bp; negative RP-14442 + RP-14398
	PR-14398	cacgcgaugttagaagcaattggagaag	

* Marked in orange are USER cloning overhangs, black are the 20 nt CRISPR recognition sites.

Supplementary list 3: List of genetic elements used in the study - Konzock, Zaghen, Norbeck 2021

	Name	YALI0	YALI1	Sequence
Promoter	pTEF1			AGAGACCGGGTTGGCGGCGTATTTGTGTCCCAAAAAACAGCCCCAATTGCCCCAATTGACCCCAAATTGACCCAGTAGCGGGCCCAAC<br>CCCGGCGAGAGCCCCCTTCACCCCACATATCAAACCTCCCCCGGTTCCCACACTTGCCGTTAAGGGCGTAGGGTACTGCAGTCTGGAA<br>TCTACGCTTGTTCAGACTTTGTACTAGTTTCTTTGTCTGGCCATCCGGGTAACCCATGCCGGACGCAAAATAGACTACTGAAAATTTTTT<br>TGCTTTGTGGTTGGGACTTTAGCCAAGGGTATAAAAGACCACCGTCCCCGAATTACCTTTCCTCTTCTTTTCTCTCTCTCCTTGTCAACTC<br>ACACCCGAAATCGTTAAGCATTTCCTTCTGAGTATAAGAATCATTCAAA
	pTef1in			AGAGACCGGGTTGGCGGCGTATTTGTGTCCCAAAAAACAGCCCCAATTGCCCCAATTGACCCCAAATTGACCCAGTAGCGGGCCCAAC<br>CCCGGCGAGAGCCCCCTTCACCCCACATATCAAACCTCCCCCGGTTCCCACACTTGCCGTTAAGGGCGTAGGGTACTGCAGTCTGGAA<br>TCTACGCTTGTTCAGACTTTGTACTAGTTTCTTTGTCTGGCCATCCGGGTAACCCATGCCGGACGCAAAATAGACTACTGAAAATTTTTT<br>TGCTTTGTGGTTGGGACTTTAGCCAAGGGTATAAAAGACCACCGTCCCCGAATTACCTTTCCTCTTCTTTTCTCTCTCTCCTTGTCAACTC<br>ACACCCGAAATCGTTAAGCATTTCCTTCTGAGTATAAGAATCATTCAAA**ATG**GTGAGTTTCAGAGGCAGCAGCAATTGCCACGGGCTT<br>TGAGCACACGGCCGGGTGTGGTCCCATTCCCATCGACACAAGACGCCACGTCATCCGACCAGCACTTTTTGCAGTACTAACCGCAG<br>(continue with 2nd AA of ORF)
	pPYK1			TGCGCGCTTCTGTTGTTTGCAACCAGTCTCCGTGGTGTGCAGCATACATTGTTCCCGCCTCTCCTTGTCTTGTTGGAAGGCCGATGTCGC<br>TGACTGTATGTACCGTTTTTTTTGTACCGTAGTACATGCAGGGCTTGGTATTTTCCAACTACAGTACATACAGGTCTTAGAGTGCTGATT<br>GGAGATAGATATGAATGGAGTGTACGAGTGGAAACAAAGCGGGTTAGATATGTGTACTTGTACATCTGTGATATTGGTAGTATTGAC<br>AAGCGGTAGTCATTTCAGTGCATCGCCGTGCCCTTTCTACTATCCCCTTGCGCCATCAATCTCCCCCTTCATCAATCCACCTCTGGCAGC<br>TCTTCTAGAAGACCTTTTTACAGTCTCCCAATTTTATCGTCTAGTGACGGCAGACCTTGTAAGCAGATATGTATCATGAGTCACGATAG<br>CTGGACAGACCAATGGCATGCGGGCAAATAACTCCCACAGACGCTCTCCCTCCGGCGCACAAAGCCTCGTGCTCTGAACACGCCCCAG<br>TTGATTTGACAGCTCTCAACATTCGTGTGAACTTTTTTAGCGGGAAAAAGTAACATGACGTTGACCGTGCGGGGCTACATGTAGCAGC<br>TGGGTGTGCTAACTACGGATACATGCCTACAACCCCCACAAGTCAAGACCATTGCGACGCGGAAACAGGAGCCCGCAAAAGAGGAGA<br>AAAACAACGGCGAGACTCGGGGGCGGAGTGGGTCACGTGACTTTCCTTTTTCCCCTCACCTGGCCCGCTCCGTCCATATCTCTGTCGTA<br>CAAGACAATATTGTCGCAACGCAAAAGGTCCATAAATTACTGGGTAGACGCAACTCTATTTGAAGGCAACCTACCGTTTGCTTTTAGTG<br>TTTTGGTTTTGTTACCATATCCAAAAAAAAACCATATATCCAAAAATTCCGCTGCACCATCTCTTCTTCTCTCCATCAACTACCCCTGCGG<br>AGAAATTCACACCACAGTTACA
	pGAPDH			GGTTGAAATGAATCGGCCGACGCTCGGTAGTCGGAAAGAGCCGGGACCGGCCGGCGAGCATAAACCGGACGCAGTAGGATGTCCTG<br>CACGGGTCTTTTTGTGGGGTGTGGAGAAAGGGGTGCTTGGAGATGGAAGCCGGTAGAACCGGGCTGCTTGGGGGGATTTGGGGCCG<br>CTGGGCTCCAAAGAGGGGTAGGCATTTCGTTGGGGTTACGTAATTGCGGCATTTGGGTCCTGCGCGCATGTCCCATTGGTCAGAATTA<br>GTCCGGATAGGAGACTTATCAGCCAATCACAGCGCCGGATCCACCTGTAGGTTGGGTTGGGTGGGAGCACCCCTCCACAGAGTAGAG<br>TCAAACAGCAGCAGCAACATGATAGTTGGGGGTGTGCGTGTTAAAGGAAAAAAAAAGAAGCTTGGGTTATATTCCCGCTCTATTTAG<br>AGGTTGCGGGATAGACGCCGACGGAGGGCAATGGCGCCATGGAACCTTGCGGATATCGATACGCCGCGGCGGACTGCGTCCGAACC<br>AGCTCCAGCAGCGTTTTTTCCGGGCCATTGAGCCGACTGCGACCCCGCCAACGTGTCTTGGCCCACGCACTCATGTCATGTTGGTGTTG<br>GGAGGCCACTTTTTAAGTAGCACAAGGCACCTAGCTCGCAGCAAGGTGTCCGAACCAAAGAAGCGGCTGCAGTGGTGCAAACGGGG<br>CGGAAACGGCGGGAAAAAGCCACGGGGGCACGAATTGAGGCACGCCCTCGAATTTGAGACGAGTCACGGCCCCATTCGCCCGCGCA<br>ATGGCTCGCCAACGCCCGGTCTTTTGCACCACATCAGGTTACCCCAAGCCAAACCTTTGTGTTAAAAAGCTTAACATATTATACCGAAC<br>GTAGGTTTGGGCGGGCTTGCTCCGTCTGTCCAAGGCAACATTTATATAAGGGTCTGCATCGCCGGCTCAATTGAATCTTTTTTCTTCTTC<br>TCTTCTCTATATTCATTCTTGAATTAAACACACATCAACA

**Terminator**	tPEX20			AAGTGTGGATGGGGAAGTGAGTGCCCGGTTCTGTGTGCACAATTGGCAATCCAAGATGGATGGATTCAACACAGGGATATAGCGAG<br>CTACGTGGTGGTGCGAGGATATAGCAACGGATATTTATGTTTGACACTTGAGAATGTACGATACAAGCACTGTCCAAGTACAATACTA<br>AACATACTGTACATACTCATACTCGTACCCGGGCAACGGTTTCACTTGAGTGCAGTGGCTAGTGCTCTTACTCGTACAGTGTGCAATAC<br>TGCGTATCATAGTCTTTGATGTATATCGTATTCATTCATGTTAGTTGCGT
	tLIP2			CTTCTGTTCGGAATCAACCTCAAGGTTAACGGCCACGATCCCCTCGTTGTTACTCTTGGTCAGCCCATTGTCGGTAACGCTGGCTTTGCT<br>AACTGGGTCGATAAACTCTTCTTTGGCCAGGAGAACCCCGATGTCTCCAAGGTGTCCAAAGACCGAAAGCTCTACCGAATCACCCACC<br>GAGGAGATATCGTCCCTCAAGTGCCCTTCTGGGACGGTTACCAGCACTGCTCTGGTGAGGTCTTTATTGACTGGCCCCTGATCCACCCT<br>CCTCTCTCCAACGTTGTCATGTGCCAGGGCCAGAGCAATAAACAGTGCTCTGCCGGTAACACTCTGCTCCAGCAGGTCAATGTGATTG<br>GAAACCATCTGCAGTACTTCGTCACCGAGGGTGTCTGTGGTATCTAAGCTATTTATCACTCTTTACAACTTCTACCTCAACTATCTACTTT<br>AATAAATGAATATCGTTTATTCTCTATGATTACTGTATATGCGTTCCTCTAAGACAAATCGAAACCAGCATGCGATCGAATGGCATACA<br>AAAGTTTCTTCCGAAGTTGATCAATGTCCTGATAGTCAGGCAGCTTGAGAAGATTGACACAGGTGGAGGCCGTAGGGAACCGATCAA<br>CCTGTCTACCAGCGTTACGAATGGCAAATGACGGGTTCAAAGCCTTGAATCCTTGCAATGGTGCCTTGGATACTGATGTCACAAACTTA<br>AGAAGCAGCCGCTTGTCCTCTTCCTCGAAACTCTCAAACACAGTCCAGAAGTCCTTTATAGTTTGATCTGTATCCAGATAGCCTCCGTA<br>ATTGGTGTGTGTCTTCAAATCCCAGACGTCCACATTGGCATGTCCTCCACTGATAAGCATTTGAAGTTCATCTGCGTTGAACATTGAGA<br>CCCACGAAGGGTCAATGAGCTGGTATAGACCGCCCAAGAATGCATCTG
	tPEX16			GCGATTGATGATTGGAAACACACACATGGGTTATATCTAGGTGAGAGTTAGTTGGACAGTTATATATTAAATCAGCTATGCCAACGGT<br>AACTTCATTCATGTCAACGAGGAACCAGTGACTGCAAGTAATATAGAATTTGACCACCTTGCCATTCTCTTGCACTCCTTTACTATATCT<br>CATTTATTTCTTATATACAAATCACTTCTTCTTCCCAGCATCGAGCTCGGAAACCTCATGAGCAATAACATCGTGGATCTCGTCAATAGA<br>GGGCTTTTTGGACTCCTTGCTGTTGGCCACCTTGTCCTTGCTGTCTGGCTCATTCTGTTTCAACGCCTTTCGCGCCAGACCATCAACCTT<br>GTTGAGCTCTCCGTCAGCAGCCTCGACCAGATCATCAAAACCAGAACCCTTGGCTCGAGTTCGGGCTTCTCGAAGCTT

**Gene**	XDH	YALI0E12463g	YALI1_E15452g	ATGTCTTCTAACCCGTCATTTGTTCTTCGAAAGCCATTGGATCTCGTCTTTGAGGATCGGCCCGACCCCAAGATCCAGGACCCCCACTCC<br>GTCAAGGTGGCAGTCAAAAAGACCGGAGTTTGCGGCTCGGATGTCCACTACTATCTGCATGGAGGAATCGGCGACTTCATTGTCAAG<br>GCTCCCATGGTTCTAGGCCATGAAAGTGCCGGAGAGGTGGTTGAGGTTGGTCCTGAAGTCAAGGACCTCAAGGTGGGAGATCGAGT<br>GGCTCTCGAGCCCGGAGTGCCGTCTCGATTGTCACAGGAGTACAAGGAGGGACGATACAACCTGTGTCCTTGCATGGTGTTTGCTGCC<br>ACCCCTCCCTACGACGGTACTCTGTGTCGTCACTACATCATTCCCGAGGACTTTTGTGTCAAGCTGCCTGATCATGTGTCTCTCGAGGA<br>GGGAGCTCTTGTGGAGCCTCTGTCCGTGGCTGTCCACTGCAACAAGCTGGCCAAGACCACTGCCCAGGACGTGGTTATTGTATTTGGA<br>GCTGGCCCAGTCGGACTGCTAGCCGTGGGAGTGGCCAATGCCTTTGGATCATCTACCATTGTGTGTGTTGATCTTGTTCCCGAGAAGC<br>TGGAGCTCGCCAAGAAGTTCGGTGCCACTCATACGTTTGTACCCACTAAGGGAGACAGTCCCAACGAGTCTGCTGACAAGATCCGAGC<br>TCTGATCAAGGGCGCTGGTCTCTCTGACTCGCCCAATGTGGCTTTGGAGTGCACCGGAGCTGAGCCTTCTATTCAGACTGCTGTTTCTG<br>TGCTGGCCACTTCCGGTCGACTTGTGCAGGTCGGCATGGGCAAGGATGACGTCAACTTCCCTATCACCAAATGCATTGTAAAGGAGAT<br>TACCGTGCTCGGATCGTTCCGATACTGCCATGGTGACTATCCCCTGGCTGTTCAGCTGGTTGCTTCTGGCAAGATTGACGTCAAGAAGC<br>TGGTGACCAACCGGTTCACCTTCAAGGAGGCTGAGCAGGCGTACAAGACGGCGGCCGAGGGCAAGGCCATCAAGATCATCATTGAC<br>GGTCCCGAGGAGGAGTAG
	XK	YALI0F10923g	YALI1_F14583g	ATGTATCTCGGACTGGATCTTTCGACTCAACAGCTCAAGGGCATCATTCTGGACACAAAAACGCTGGACACGGTCACACAAGTCCATG<br>TGGACTTTGAGGACGACTTGCCGCAGTTCAACACCGAAAAGGGCGTCTTTCACAGCTCTACAGTGGCCGGAGAAATCAATGCTCCTGT<br>GGCAATGTGGGGGGCAGCTGTGGACTTGCTGATAGAGCGTCTGTCAAAGGAAATAGACCTTTCCACGATCAAGTTTGTGTCGGGCTC<br>GTGCCAGCAACACGGCTCTGTTTATCTCAACAGCAGCTACAAGGAGGGCCTGGGTTCTCTGGACAAACACAAAGACTTGTCTACAGGA<br>GTGTCATCCTTACTGGCGCTCGAAGTCAGCCCCAATTGGCAGGATGCAAGCACGGAGAAGGAGTGTGCGCAGTTTGAGGCTGCAGTC<br>GGCGGTCCCGAGCAGCTGGCTGAGATCACTGGCTCTCGAGCACATACTCGTTTCACCGGGCCCCAGATTCTCAAGGTCAAGGAACGC<br>AACCCCAAGGTATTCAAGGCCACGTCACGGGTCCAGCTCATATCCAACTTTCTAGCATCTCTGTTTGCCGGCAAGGCGTGCCCCTTTGA<br>TCTTGCTGACGCCTGTGGAATGAATCTGTGGGACATCCAGAATGGCCAGTGGTGCAAGAAACTCACAGATCTCATCACCGATGACACC<br>CACTCGGTCGAGTCCCTCCTTGGAGACGTGGAAACAGACCCCAAGGCTCTACTGGGCAAAATCTCGCCCTATTTCGTCTCCAAGGGCT<br>TCTCTCCCTCTTGTCAGGTGGCACAGTTCACAGGCGACAACCCAGGCACTATGCTGGCTCTCCCCTTACAGGCCAATGACGTGATTGTG<br>TCTTTGGGAACATCTACGACCGCCCTCGTCGTAACAAACAAGTACATGCCCGACCCCGGATACCATGTGTTCAACCACCCCATGGAGG<br>GATACATGGGCATGCTGTGCTACTGCAACGGAGGTCTAGCACGAGAGAAGATCCGAGACGAGCTTGGAGGCTGGGACGAGTTTAAT<br>GAGGCGGCCGAGACCACCAACACAGTGTCTGCTGACGATGTCCATGTTGGCATCTACTTTCCACTACGAGAAATCCTTCCTCGAGCAG<br>GTCCCTTTGAACGACGTTTCATCTACAACAGACAAAGTGAACAGCTTACAGAGATGGCTTCTCCAGAGGACTCACTGGCAACCGAACA<br>CAAACCGCAGGCTCAAAATCTCAAGGACACGTGGCCGCCACAAATGGACGCCACTGCCATCATTCAAAGCCAGGCCCTCAGTATCAAA<br>ATGAGACTCCAACGCATGATGCATGGCGATATTGGAAAGGTGTATTTTGTGGGAGGCGCCTCGGTCAACACTGCTATCTGCAGCGTAA<br>TGTCTGCCATCTTAAAACCAACAAAGGGCGCTTGGAGATGTGGTCTGGAAATGGCAAACGCTTGTGCCATTGGAAGTGCCCATCACGC<br>CTGGCTTTGCGACCCCAACAAGACAGGCCAGGTACAGGTTCACGAAGAAGAGGTCAAATACAAGAATGTGGACACAGACGTGCTACT<br>CAAGGCGTTCAAGCTGGCCGAAAACGCCTGCCTGGAGAAATAA

**Gene**	XR	YALI0D07634g	YALI1_D09870g	ATGTCCTTCAAGCTCGCCTCCGGAAAGTCCATGCCCAAGGTCGGATTCGGCCTGTGGAAGGTCCCCGTGACAAGACCGCCGACACCG<br>TCTACGGAGCCATCAAGAACGGTTACAGACTGTTTGACGGCGCCTTCGACTACCAGAACGAGCGAGAGGCCGGCGAAGGTATCCGAC<br>GAGCCATCAAGGATGGCCTGGTCAAGCGAGAGGACATCTTCATCACCACCAAGTTGTGGAACACCTTCCACTCAAAGGAGCACGCTCT<br>GCAGATCGCCAAGGAGCAGAACGAGTGGTGGGGACTCGACTACATCGATCTCTACCTCATCCACTTCCCCATCCCCATGCAGTACATT<br>CCCATCTCCGAGAAGGAGTGGGCTGGATGGACCAACGCCACTGACTCGGGTCCTAACCCTCTGGCCAAGATCCCTACCCGAGAGACCT<br>GGGAGGCTCTCGAGGAGCTAGTTGATACCGGAATCGCCAAGTCCATTGGTGTCTCCAACTTCACCGCCCAGAACATTTACGACGTCCA<br>GACCTACAACAAGCACCCCATTTCTGCTCTGCAGATTGAGCACCACCCCTACCTGGTGCAGCCCCAGCTGACCCAGCTCGCAAAGGAC<br>AACAACATCCAGGTCACTGCCTACTCTTCTTTCGGCCCCGCCTCCTTTGTGGAGATTGGCATGGACCAGAAGGTCCCTCCTCTTTTCGA<br>GAACGAGACCATCACCAAGATCGCCAAGGCTCACAACAAGACCCCCTCCCAGGTTCTGCTGCGATGGGCTACCCAGCGAGGCATTGC<br>CGTCATCCCCAAGTCCAACAACGTCGAGCGACAAACTCAGAACCTCGAATCTCTGGACTTTGACCTGACCGAGGCCGAGATTAAGGAG<br>ATCTCCAACCTCAACAAGAACCTGCGATTCAACGATCCCGGTGTCTACGCTAACCTTCCCATTTTCGCCTAA
	DGA1	YALI0E32769g	YALI1_E38810g	(ATG)ACTATCGACTCACAATACTACAAGTCGCGAGACAAAAACGACACGGCACCCAAAATCGCGGGAATCCGATATGCCCCGCTATCG<br>ACACCATTACTCAACCGATGTGAGACCTTCTCTCTGGTCTGGCACATTTTCAGCATTCCCACTTTCCTCACAATTTTCATGCTATGCTGCG<br>CAATTCCACTGCTCTGGCCATTTGTGATTGCGTATGTAGTGTACGCTGTTAAAGACGACTCCCCGTCCAACGGAGGAGTGGTCAAGCG<br>ATACTCGCCTATTTCAAGAAACTTCTTCATCTGGAAGCTCTTTGGCCGCTACTTCCCCATAACTCTGCACAAGACGGTGGATCTGGAGC<br>CCACGCACACATACTACCCTCTGGACGTCCAGGAGTATCACCTGATTGCTGAGAGATACTGGCCGCAGAACAAGTACCTCCGAGCAAT<br>CATCTCCACCATCGAGTACTTTCTGCCCGCCTTCATGAAACGGTCTCTTTCTATCAACGAGCAGGAGCAGCCTGCCGAGCGAGATCCTC<br>TCCTGTCTCCCGTTTCTCCCAGCTCTCCGGGTTCTCAACCTGACAAGTGGATTAACCACGACAGCAGATATAGCCGTGGAGAATCATCT<br>GGCTCCAACGGCCACGCCTCGGGCTCCGAACTTAACGGCAACGGCAACAATGGCACCACTAACCGACGACCTTTGTCGTCCGCCTCTG<br>CTGGCTCCACTGCATCTGATTCCACGCTTCTTAACGGGTCCCTCAACTCCTACGCCAACCAGATCATTGGCGAAAACGACCCACAGCTG<br>TCGCCCACAAAACTCAAGCCCACTGGCAGAAAATACATCTTCGGCTACCACCCCCACGGCATTATCGGCATGGGAGCCTTTGGTGGAA<br>TTGCCACCGAGGGAGCTGGATGGTCCAAGCTCTTTCCGGGCATCCCTGTTTCTCTTATGACTCTCACCAACAACTTCCGAGTGCCTCTCT<br>ACAGAGAGTACCTCATGAGTCTGGGAGTCGCTTCTGTCTCCAAGAAGTCCTGCAAGGCCCTCCTCAAGCGAAACCAGTCTATCTGCAT<br>TGTCGTTGGTGGAGCACAGGAAAGTCTTCTGGCCAGACCCGGTGTCATGGACCTGGTGCTACTCAAGCGAAAGGGTTTTGTTCGACTT<br>GGTATGGAGGTCGGAAATGTCGCCCTTGTTCCCATCATGGCCTTTGGTGAGAACGACCTCTATGACCAGGTTAGCAACGACAAGTCGT<br>CCAAGCTGTACCGATTCCAGCAGTTTGTCAAGAACTTCCTTGGATTCACCCTTCCTTTGATGCATGCCCGAGGCGTCTTCAACTACGAT<br>GTCGGTCTTGTCCCCTACAGGCGACCCGTCAACATTGTGGTTGGTTCCCCCATTGACTTGCCTTATCTCCCACACCCCACCGACGAAGA<br>AGTGTCCGAATACCACGACCGATACATCGCCGAGCTGCAGCGAATCTACAACGAGCACAAGGATGAATATTTCATCGATTGGACCGA<br>GGAGGGCAAAGGAGCCCCAGAGTTCCGAATGATTGAGTAA
